# A Novel Endosurgical Prosthesis to Treat Thoracoabdominal Aortic Aneurysm in Complex Anatomy or Emergency Settings

**DOI:** 10.1055/s-0040-1702143

**Published:** 2020-07-31

**Authors:** Guglielmo Saitto, Antonio Scafuri, Saimir Kuci, Alfred Ibrahimi, Jacob Zeitani

**Affiliations:** 1 Department of Cardiac Surgery, Istituto di ricovero e cura a carattere scientifico, San Donato Milanese Hospital, San Donato Milanese, Italy; 2 Department of Cardiac Surgery, Tor Vergata University of Rome, Rome, Italy; 3 Department of Anesthesiology, Reanimation Mother Teresa University, Tirana, Albania; 4 Department of Biomedical Sciences and Specialized Surgery, University of Ferrara, Ferrara, Italy

**Keywords:** thoracoabdominal aortic aneurysm, endovascular approach, hybrid prosthesis

## Abstract

**Background** Despite improvements in operative techniques, open thoracoabdominal aortic aneurysm (TAAA) repair is complex and characterized by high mortality and morbidity rate. Less invasive techniques have been developed since 2005 for the treatment of TAAA. Unfortunately, many of these devices require custom fabrication, resulting in delay of many weeks until treatment can be delivered but crucial in critical emergency cases. We present a novel hybrid endovascular and surgical prosthesis, which was tested on five pigs, with the aim of reducing the barrier issues of endovascular therapy in such particular cases.

**Methods** The principal characteristic of the proposed hybrid endovascular prosthesis is to combine a proximal and distal stented zones and, in between, a classical surgical blood tied Dacron prosthesis. The device was tested in five pigs where feasibility of implantation and acute postoperative outcomes were evaluated, including bleeding, bowel ischemia, renal function, and peripheral blood perfusion.

**Results** In all cases, following laparotomy, the endoprosthesis was successfully implanted under fluoroscopy and the surgical prosthesis zone could be easily detected by the radio-opaque markers. No major bleeding or cardiac events occurred throughout preparation and implantation. One hour after prosthesis implantation and surgical anastomoses of all vessels were completed, normal urine output was registered, and no acidosis was detected.

**Conclusions** This novel graft has shown ease of endoprosthesis and visceral vessels implantation without the need of thoracotomy or extracorporeal circulation and may be useful in an emergency setting or high risk and complex anatomy TAAA unsuitable for traditional endovascular aneurysm repair, or to avoid an excess waiting time for a “custom made” prosthesis. The great adaptability of this “hybrid” prosthesis in complex anatomy for the majority of TAAA could be important in high-risk patients and in some difficult situations, such as a high risk of imminent rupture.

## Introduction

Despite improvements in operative technique and anesthetic support, open thoracoabdominal aortic aneurysm (TAAA) repair remains complex and characterized by high mortality and morbidity rates.

The risk of postoperative death or complications is not only increased in the highest anatomical complexity TAAA cases (Crawford II) but also in certain patient populations, such as older patients and those with congestive heart failure, poor pulmonary function, or renal disease. Paraplegia, paraparesis, renal failure, stroke, and intestinal ischemia represent some of the most serious complications related to aortic manipulation or distal aortic ischemia. 1

Because of the added operative risk in patients with these comorbidities, less invasive techniques have been developed. In 2005, the first thoracic endograft was approved by U.S. Food and Drug Administration for the treatment of TAAA. By avoiding thoracotomy, extracorporeal perfusion, aortic crossclamping, and single lung ventilation, the hybrid TAAA procedure has been suggested to have decreased mortality in high-risk patients. 2 Combined open and endovascular hybrid TAAA repair generally involves one or two stages to accomplish TEVAR (thoracic endovascular aortic repair) in these cases. The construction of extra-anatomical bypass to either visceral or arch vessels is often performed.

In a recent large meta-analysis, the authors considered outcomes of 528 hybrid TAAA repairs, reporting a high mortality rate of 14.3% and substantial complication rates (7.0% for spinal cord ischemia, 4.5% for mesenteric ischemia, and 7.0% for permanent renal failure). 3 However, in other publications, mesenteric ischemia after hybrid TAAA repair was more prevalent, ranging from 17 to 40%. The length and angulation of the superior mesenteric artery bypass graft were found to be predictors of these complications. 4

Successful total endovascular approaches to TAAA include repair using fenestrated devices or grafts with branches to accommodate the visceral arteries and other alternative surgical approaches have been proposed to include parallel, snorkel, telescope, and chimney grafts. 5 Unfortunately, many of these off-label devices require custom fabrication, resulting in delay of weeks until treatment can be delivered: most custom-made devices have about 6-week manufacturing delay. Moreover, these custom-made devices can be very expensive and the long-term results are still scarce. 6

For these reasons, in patients with highly complex anatomy or emergency settings, it is often difficult to maintain a total endovascular approach to TAAA. We present a novel hybrid endovascular and surgical prosthesis (conceived by J.Z. and produced by Jotec GmbH, Hechingen, Germany) which was tested in five pigs with the aim of reducing the barrier issues of endovascular therapy in thoracoabdominal cases.

## Materials and Methods

### The Novel “Hybrid” Prosthesis

**Fig. 1:**
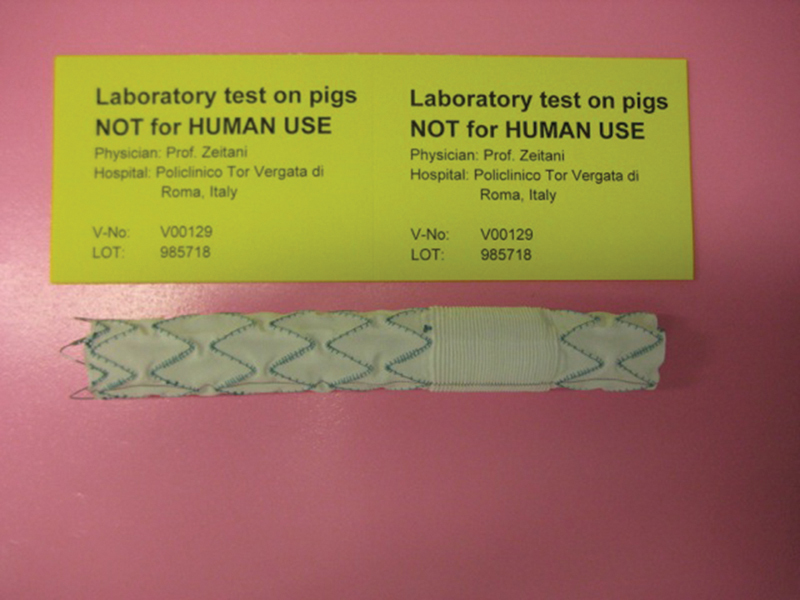
The novel hybrid prosthesis, with a proximal and distal endovascular zone and in between a classical surgical prosthesis.

The principal characteristic of this hybrid prosthesis is to combine proximal and distal endovascular-stented zones and in between classical surgical blood-tied Dacron prosthesis. The surgical zone identification following positioning under fluoroscopy is simplified by radio opaque markers. The device is crimped into a delivery system as conventional vascular endoprosthesis. The prosthesis is shown in Fig. 1 . Following engineering development, the prosthesis was tested in the laboratory. Acute animal tests were performed on five domestic female pigs with a mean bodyweight of 100 ± 2 kg. Experiments were performed at the University Heart Centre of Naples, Italy, under supervision and assistance of a veterinary team. The animal study was directed according to the European Guidelines for the Protection of Animals (86/609/EEC).

### Technical Procedure

Surgical procedures were performed under general anesthesia after orotracheal intubation. In all cases a median laparotomy provided the surgical access to abdominal aorta.

Anticoagulation was achieved with heparin (400 IU/kg BW) to activate clotting time of at least 300 seconds. Two 6G sheaths were inserted into the right carotid and femoral arteries to monitor the mean arterial blood pressure proximally and distally to the abdominal aorta and for arterial blood gas analyses. All hemodynamics and respiratory parameters were continuously monitored and recorded according to a standardized protocol during each intervention.

**Fig. 2:**
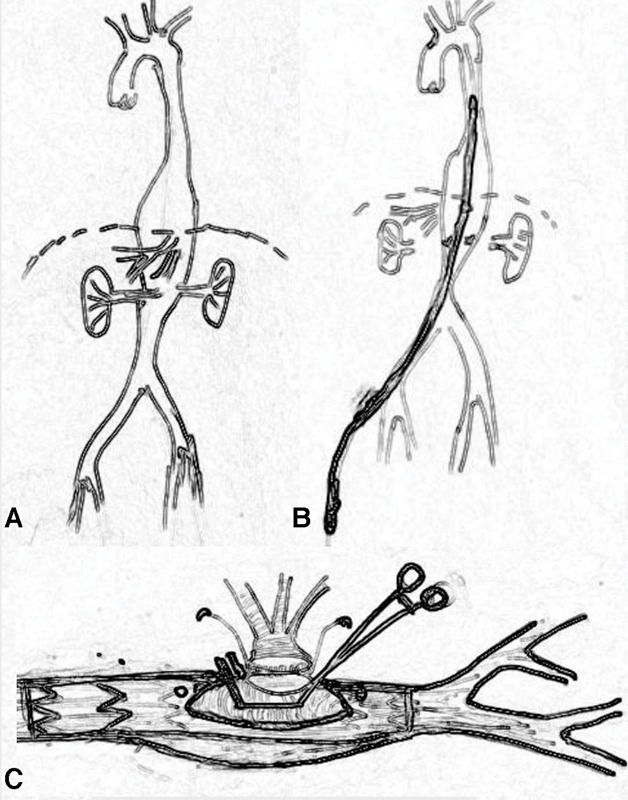
( **A** ) Step-by-step illustrations of the procedure: thoracoabdominal aortic aneurysm. ( **B** ) Insertion of the prosthesis through the right femoral artery and correct positioning and release of the endoprosthesis. ( **C** ) Surgical opening of the aortic aneurysm with the exposure of the Dacron “not stented” segment of the prosthesis and partial clamp to complete the anastomosis in an end-to-side fashion of a multibranch Dacron graft for the reimplantation of the main visceral vessels (celiac trunk, superior mesenteric, and renal arteries).

After median laparotomy and visceral arteries exposure, heparin was given. Using a stiff guidewire, positioned under fluoroscopy in the descending aorta via the right iliac artery, the endoprosthesis, loaded in a 20-Fr delivery system, was introduced into the vessel and deployed under C-arm fluoroscopy control. In particular, during deployment, it is important to identify the surgical zone in the abdomen aorta by the radio opaque markers to enable graft to graft anastomosis. The celiac, superior mesenteric, and renal arteries were detached from the aorta. In correspondence to the surgical prosthesis segment, the aortic wall was then incised exposing the Dacron not stented prosthesis. A partial clamp is positioned, the Dacron graft is incised, and a multibranch Dacron graft is anastomosed with a running 5–0 polypropylene suture to enable all visceral vessels anastomosis in an end-to-end fashion. Compliant with the aortic anatomy, renal arteries might be anastomosed separately in an end-to-end fashion while the celiac and superior mesenteric arteries might be anastomosed together as one “bottom” to one side Dacron graft branch to reduce surgical times. Step by step illustrations are shown in Fig. 2A–C .

1. Correct insertion positioning and release of the endoprosthesis under C-arm fluoroscopy ( Fig. 2B ).
2. Exposure of the Dacron graft, partial aortic clamp at the surgical prosthesis segment allowing multibranch Dacron graft anastomosis in an end to side fashion ( Fig. 2C ).

At the end of the experiments, the pigs were sacrificed by intravenous injection of T61 under deep anesthesia.

## Results

Under fluoroscopy, graft deployment was successful, and radio-opaque markers were useful to identify and position the surgical segment at the correct thoracic and abdominal aorta height in all five animals. Once the abdominal aorta was incised and the surgical prosthesis zone was exposed to enable partial crossclamping, no bleeding was noted from the exposed surgical field. At the end of the surgical procedure, no major bleeding or cardiac events occurred.

**Fig. 3:**
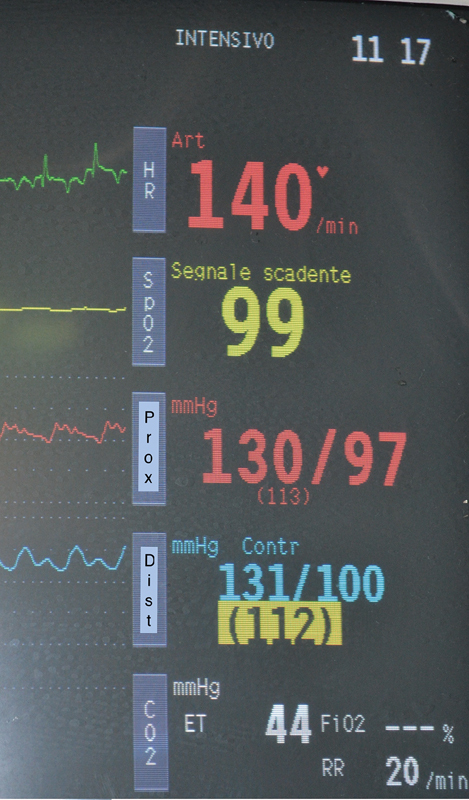
Invasive blood pressure at the end of the hybrid procedure to show the same arterial pressure in the proximal and distal part of the prosthesis.

No differences were registered with regard to blood pressure at the distal artery in comparison to the preoperative values ( Fig. 3 ). One hour after the anastomosis was complete normal urine output was registered and no acidosis was detected.

## Discussion

The thoracoabdominal aneurysm is a complex vascular disease and can be treated with reasonable results only in referral centers with highly skilled surgeons, anesthesiologists, and perfusion technicians. 7 The increasing number of endovascular procedures and experience to treat diseased aorta, better materials, and custom-made solutions enable treating aneurysms involving the descending aorta. However, although less traumatic, also for this therapeutic solution skilled physicians in vascular and interventional are required. The custom-made graft, produced on specific patient’s vascular anatomy, requires time before delivery and as so, is not “on the shelf graft”; it may happen that the patients who are already waiting for the personalized endoprosthesis graft will experience aorta rupture and will require emergency treatment. A recent article shows how, in complex aortic aneurysm, clinically relevant differences of several important in-hospital outcomes were found in the use of custom made stent grafts versus physician-modified fenestrated grafts. 8 The combined one step endovascular and surgical procedure is not a new concept and is being practiced in different settings, especially when emergency intervention is required where the vascular anatomy and/or clinical conditions are not favorable for any of the options alone. 9

### Potential Impact on Daily Practice

This experimental study was designed to test technical feasibility of hybrid repair of the thoracoabdominal aorta using a novel device. The prototype of the device consists of a proximal and distal stent graft attached to a central surgical blood-tied Dacron portion. The latter is marked with radio-opaque markers, allowing correct positioning. The aim of the design of this prosthesis is to reduce operative morbidity and complications related to the conventional surgical approach. Indeed, we could demonstrate the ease of endoprosthesis and visceral vessels implantation without the need of thoracotomy or extracorporeal circulation.

Following the native aorta incision to expose the surgical prosthesis zone, no bleeding around the prosthesis occurred, obviously the nondilated aorta in the experimental setting was in favor. In real surgical aneurysm setting, endoleak type one or two might have occurred, especially the latter where the thoracic and lumbar arteries arose at the nonstented zone. In this scenario, a running polypropylene suture attaching the free native aortic wall to the Dacron prosthesis should be performed. In the experimental setting, all vessels were detached from the aorta and anastomosed in an end-to-end fashion; however, based on vessel's location, alternatively, the vessels can be ligated and anastomosis end-to-side can be performed. Also, if aortic pathology involves the iliac artery, the distal stent graft may be removed and aorto bi-iliac anastomosis can be performed using the appropriate bifurcated Dacron graft.

The use of this prosthesis might be very helpful in an emergency setting or high-risk and complex anatomy TAAA unsuitable for traditional endovascular aneurysm repair or avoiding a too long waiting time for a “custom made” prosthesis. The great adaptability of this “hybrid” prosthesis in complex anatomy of the majority of TAAA could be of great help in high-risk patients and in some difficult situations, like patients with high risk of imminent rupture.

Also, the end-to-end anastomosis, permitting almost anatomical configuration, reduces the risk of malperfusion and thrombosis related to long extra-anatomical visceral arterial grafts.

Of course, laparotomy makes this procedure more invasive with respect to the total endovascular approach. But the surgical times are very short, avoiding aortic crossclamping, any thoracotomy, single lung ventilation, and the long extra-anatomical bypass of visceral vessels, with the attendant's high risk of thrombosis and embolism.

Of note, we performed the surgical procedure using solely C-arm fluoroscopy, in a standard operating theater, whereas a total endovascular procedure for such complex aortic pathology requires a hybrid operating room.

The determination of the best treatment approach for patients with complex thoracoabdominal aortic disease will likely be made based on the patient's health as well as the individual clinician's familiarity with the technical options. The device reported in this paper may provide another option.

## Conclusions

The experimental tests conducted in the swine model suggested that the proposed hybrid graft might be used to cover the thoracoabdominal aorta as commercialized stent grafts where the nonstented zone enables partial crossclamp and graft anastomosis in open surgery. The graft might be useful in particular in emergency settings when the thoracoabdominal aorta Crawford classification Type III–IV should be treated.

## References

[1] Aftab M Songdechakraiwut T Green S Y Contemporary outcomes of open thoracoabdominal aortic aneurysm repair in octogenarians J Thorac Cardiovasc Surg 2015 149(2, Suppl):S134 S141 25439781 10.1016/j.jtcvs.2014.09.038

[2] Zhou W Reardon M Peden E K Lin P H Lumsden A B Hybrid approach to complex thoracic aortic aneurysms in high-risk patients: surgical challenges and clinical outcomes J Vasc Surg 2006 44 04 688 693 16926086 10.1016/j.jvs.2006.06.013

[3] Moulakakis K G Mylonas S N Antonopoulos C N Liapis C D Combined open and endovascular treatment of thoracoabdominal aortic pathologies: a systematic review and meta-analysis Ann Cardiothorac Surg 2012 1 03 267 276 23977508 10.3978/j.issn.2225-319X.2012.08.02 PMC3741767

[4] Chiesa R Tshomba Y Logaldo D Kahlberg A Baccellieri D Apruzzi L Possible graft-related complications in visceral debranching for hybrid B dissection repair Ann Cardiothorac Surg 2014 3 04 393 399 25133102 10.3978/j.issn.2225-319X.2014.05.06 PMC4128930

[5] Oderich G S Ribeiro M Reis de Souza L Hofer J Wigham J Cha S Endovascular repair of thoracoabdominal aortic aneurysms using fenestrated and branched endografts J Thorac Cardiovasc Surg 2017 153 02 S32 S41.e7 27866781 10.1016/j.jtcvs.2016.10.008

[7] Locham S Hussain F Dakour-Aridi H Barleben A Lane J S Malas M Hospital volume impacts the outcomes of endovascular repair of thoracoabdominal aortic aneurysmsAnn Vasc Surg2019(e-pub ahead of print). Doi: 10.1016/j.avsg.2019.09.018 10.1016/j.avsg.2019.09.018 31629842

[8] Sénémaud J N Ben Abdallah I de Boissieu P Intraoperative adverse events and early outcomes of custom-made fenestrated stent grafts and physician-modified stent grafts for complex aortic aneurysmsJ Vasc Surg2019(e-pub ahead of print). Doi: 10.1016/j.jvs.2019.07.102 10.1016/j.jvs.2019.07.102 PMC7126501 31708298

[9] Zeitani J Mve Mvondo C Chiariello G Nardi P Chiariello L Hybrid approach to treat total thoracic aortic aneurysm in a patient undergoing emergency surgery for descending aortic rupture Thorac Cardiovasc Surg 2013 61 07 594 596 23585222 10.1055/s-0033-1333895

